# Early Postoperative Outcomes of Periprosthetic Femoral Fracture in Patients Over 90 Years of Age

**DOI:** 10.7759/cureus.57145

**Published:** 2024-03-28

**Authors:** Ryo Araki, Toru Asari, Tatsuhiro Fukutoku, Kazunari Takeuchi, Yoshihide Nakamura

**Affiliations:** 1 Department of Orthopedic Surgery, Hirosaki General Medical Center, Hirosaki, JPN

**Keywords:** walking ability, mortality, vancouver classification, super elderly patients, periprosthetic femoral fracture (pff)

## Abstract

The number of total hip arthroplasty and bipolar hemiarthroplasty is increasing because of their good clinical outcomes and the aging population. Consequently, the incidence of periprosthetic femoral fractures (PFFs) is expected to increase in older patients with osteoporosis. Surgery is the first choice of treatment for PFF, except in Vancouver Type A fractures. However, surgical treatment of PFF, including open reduction and internal fixation (ORIF) and revision arthroplasty, is highly invasive, and high mortality rates have been reported. The indication for ORIF for PFF in very elderly patients at a high risk of complications remains controversial, and postoperative outcomes are uncertain. This study aimed to evaluate the postoperative outcomes of ORIF for PFF in elderly patients. We retrospectively analyzed four females with a mean age of 90.7 years (91-92 years) who underwent ORIF for PFF at our institution from September 2014 to January 2023. No cases of American Society of Anesthesiologists (ASA) grade 3 or higher were found. Three patients were classified as Vancouver Type B1, and one was classified as Vancouver Type C. Cementless stems were used in primary surgeries in all cases. To measure clinical outcomes, we investigated the patient’s walking ability at 30 days, three months postoperatively, and the final follow-up. Mortality was assessed during the follow-up period. One patient could walk without walking aids preoperatively, two used a walking stick, and one used a walker. All patients remained hospitalized and underwent gait training with a walker at 30 days follow-up; however, at three months postoperatively and the final follow-up, no patient was unable to walk. No deaths occurred within one month of surgery. Three deaths occurred during follow-up: one within six months, one within one year, and one within five years of surgery. The postoperative ORIF results for PFF in patients aged > 90 years showed no fatal perioperative complications and low mortality within 30 days postoperatively. These results suggest that ORIF for PFF can be considered for elderly patients if the preoperative ASA grade is relatively low.

## Introduction

Total hip arthroplasty (THA) and bipolar hemiarthroplasty (BHA) are performed annually in thousands of patients, and previous studies have reported stable long-term postoperative outcomes and high survival rates [1,2]. However, periprosthetic femoral fracture (PFF) is a concern in elderly patients with osteoporosis [3]. In an aging society, the incidence of PFF is increasing because of the increase in the number of THA performed [4,5]. The incidence of PFF is 0.4%-1.1% and 2.1%-4.0% for primary and revision THA, respectively [6,7].

PFF treatment is technically demanding and can lead to poor clinical outcomes owing to complications, such as implant failure, infection, nonunion, dislocation, and aseptic loosening [8]. Vancouver Type B1 and C fractures with stable implants are indications for open reduction and internal fixation (ORIF) [9]. However, mortality within one year after surgical treatment of PFF for which ORIF is indicated is higher in patients treated with ORIF than in those treated with revision THA [10]. The indication for ORIF for PFF in very elderly patients at a high risk of complications remains controversial, and postoperative outcomes are uncertain. This study aimed to evaluate the postoperative outcomes of ORIF in four cases of PFF in very elderly patients.

## Case presentation

Between September 2014 and January 2023, 16 consecutive ORIFs of PFF were performed at our institution. We retrospectively analyzed these cases and selected patients aged >90 years. Four women had a mean age of 90.7 years (90-92 years). For all four cases, cementless stems were used in primary surgeries, and ORIF was performed using plate fixation combined with cerclage wiring. All patients underwent postoperative rehabilitation according to each protocol, including range-of-motion training of the operated hip and gait training. Informed consent was obtained from all participants. The ethics committee of our institution approved this study.

Medical records were reviewed, and the following data were collected: surgical method of primary surgery, mean follow-up period from primary surgery to injury, American Society of Anesthesiologists (ASA) grade, fracture type, operation time, blood loss, perioperative complications, time to surgery, and mean length of hospital stay. The fracture types were classified by one trauma surgeon and one hip surgeon using plain radiography according to the Vancouver Classification System [11].

To measure the clinical outcome, we investigated the patients’ walking ability (without walking aid, walking stick, walker, or wheelchair) at 30 days, three months postoperatively, and at the final follow-up. Mortality was assessed during the follow-up period. These data were collected from medical records and via telephone contact with their relatives.

**Figure 1:**
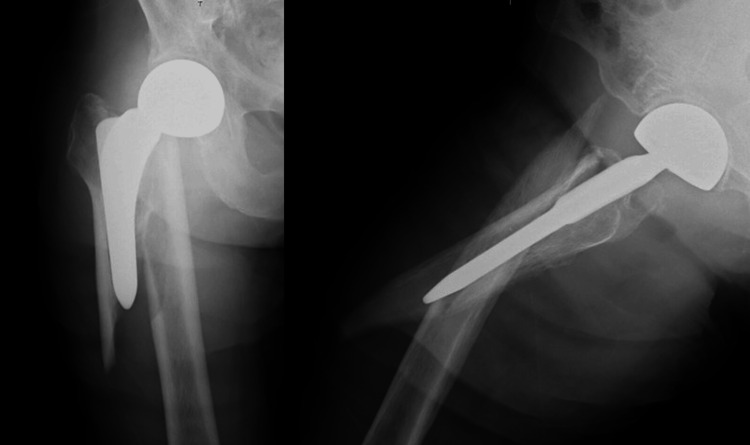
Preoperative radiographs of case 1 Preoperative radiographs of case 1 showed PFF of Vancouver classification Type B1. PFF: periprosthetic femoral fracture.

**Figure 2:**
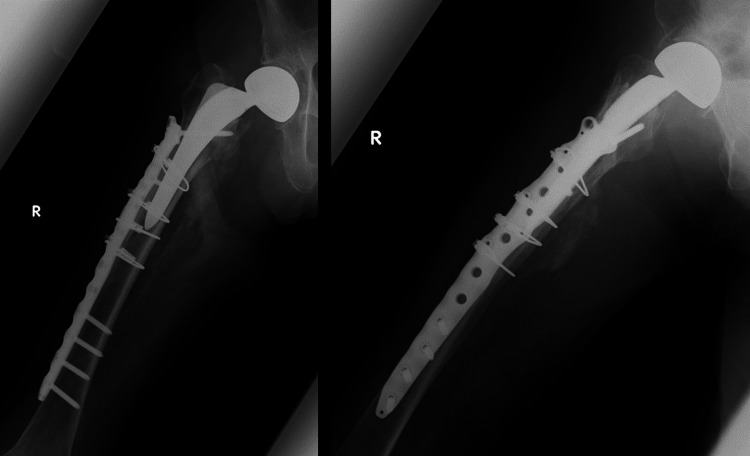
Postoperative radiographs of case 1 ORIF with plate fixation and cerclage wiring was performed. ORIF: open reduction and internal fixation.

Case 1 is a 90-year-old woman who received treatment for hypertension and dementia. She had previously undergone BHA for a femoral neck fracture. Approximately two months after the primary surgery, the patient fell and was diagnosed with a Vancouver Type B1 PFF (Figure 1). The pre-injury ASA grade was 2 (Table 1). She could walk without any walking aid before the injury. The patient required preoperative treatment for a urinary tract infection and had to wait 16 days preoperatively. Subsequently, plate fixation and cerclage wiring were performed (Figure 2). The operative time and blood loss were 131 min and 317 ml, respectively (Table 2). Postoperatively, weight-bearing was restricted for two weeks, and partial weight-bearing was allowed in the third postoperative week. Although she could not regain walking ability (Table 3), bone union was achieved at three months postoperatively, and she was still alive at three years postoperatively.

**Table 1:** Demographic data Primary surgeries included BHA in three cases and THA in one case. The mean time from primary surgery to fracture was 74.3±40.2 months. The pre-injury ASA grade was 2 in all patients. The Vancouver classification was Type B1 in three cases and Type C in one case. ASA: American Society of Anesthesiologists, BHA: bipolar hemiarthroplasty, THA: total hip arthroplasty.

Sex/Age (years)	Primary surgery	Follow-up period from primary surgery to injury (months)	ASA grade	Vancouver classification
Female/90	BHA for femoral neck fracture	2	2	B1
Female/90	BHA for femoral neck fracture	3	2	B1
Female/92	BHA for femoral neck fracture	95	2	B1
Female/91	THA for hip osteoarthritis	197	2	C

**Table 2:** Perioperative parameter Multiple blood transfusions were performed for low postoperative hemoglobin except No.2 patient. Two patients underwent surgery more than 10 days after the injury due to the preoperative urinary tract infection (UTI) and COVID-19 positive, respectively.

No	Operation time (minutes)	Blood loss (g)	Perioperative RBC transfusion volume (units)	Perioperative complications (pre-/postoperative)	Time to surgery (days)	Stay at our hospital (days)
1	131	317	8	UTI/none	16	45
2	139	124	0	None	8	40
3	107	733	10	None	4	24
4	130	697	10	COVID-19/none	14	78

**Table 3:** Pre- and postoperative mobility Although all patients received gait training with a walker at the 30-day follow-up, they could not regain practical walking ability. After discharge from the hospital, patients were unable to go to the hospital for rehabilitation and no longer had the opportunity to walk. Three months postoperatively and at the final follow-up, all patients were unable to walk.

Before injury	30-day follow-up	3 months follow-up	Final follow-up
Without walking aid	walker	wheelchair	wheelchair
Walker	walker	wheelchair	wheelchair
Walking stick	walker	wheelchair	wheelchair
Walking stick	walker	wheelchair	wheelchair

**Figure 3:**
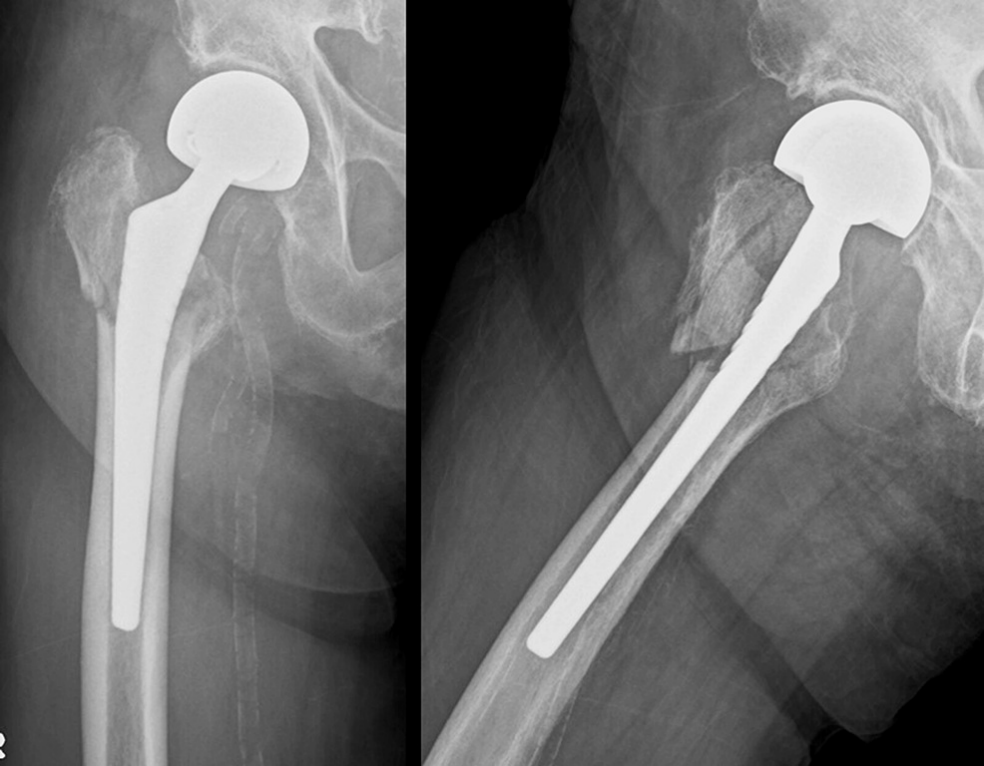
Preoperative radiographs of case 2 Preoperative radiographs of case 2 showed PFF of Vancouver classification Type B1. PFF: periprosthetic femoral fracture.

**Figure 4:**
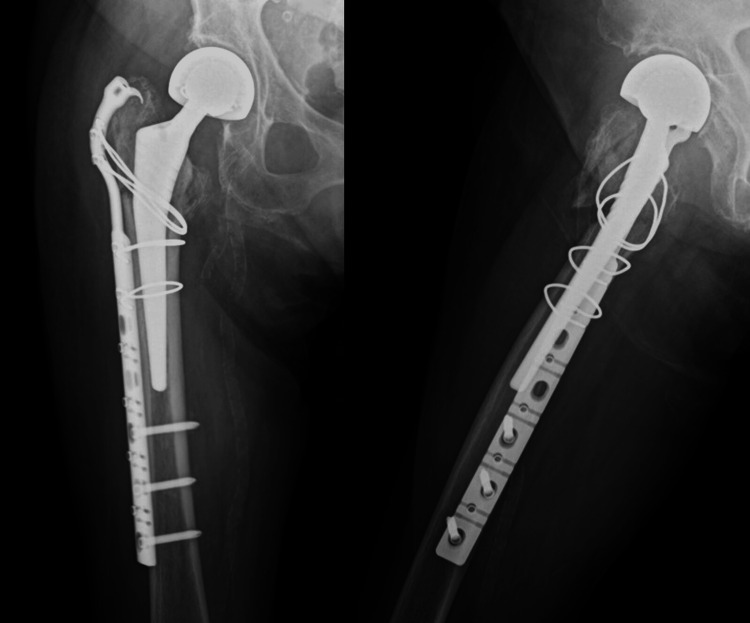
Postoperative radiographs of case 2 ORIF with plate fixation and cerclage wiring was performed. ORIF: open reduction and internal fixation.

Case 2 is a 90-year-old woman who received treatment for diabetes, hypertension, and dementia. Her blood glucose levels and blood pressure were well-controlled with medication. She had previously undergone BHA for a femoral neck fracture. Approximately four months after the primary surgery, the patient fell and was diagnosed with a Vancouver Type B1 PFF (Figure 3). The pre-injury ASA grade was 2 (Table 1). She could walk with a walker before the injury. The patient underwent ORIF combined with plate fixation and cerclage wiring on day 8 after injury (Figure 4). The operative time and blood loss were 131 min and 124 ml, respectively (Table 2). Postoperatively, weight-bearing was not restricted. The patient had no postoperative complications and underwent gait training using a walker 30 days after the surgery. However, three months postoperatively, she could not walk (Table 3) and died approximately one year postoperatively.

**Figure 5:**
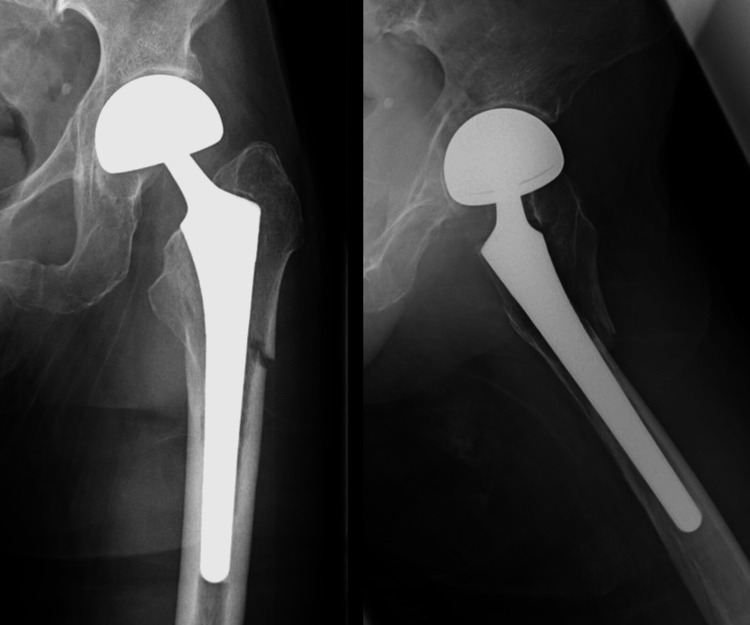
Preoperative radiographs of case 3 Preoperative radiographs of case 3 showed PFF of Vancouver classification Type B1. PFF: periprosthetic femoral fracture.

**Figure 6:**
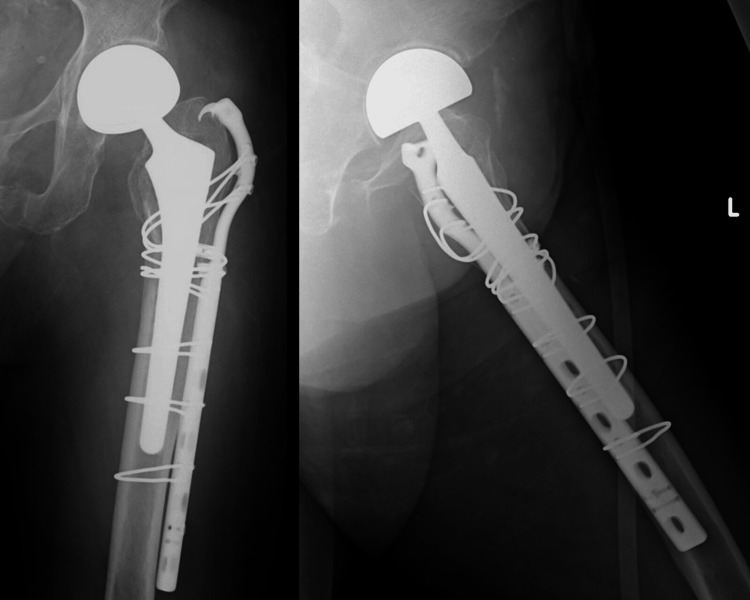
Postoperative radiographs of case 3 ORIF with plate fixation and cerclage wiring was performed. ORIF: open reduction and internal fixation.

Case 3 is a 92-year-old woman who received treatment for auricular fibrillation and dementia. She had previously undergone BHA for a femoral neck fracture. Approximately eight years after the primary surgery, the patient fell and was diagnosed with a Vancouver Type B1 PFF (Figure 5). The pre-injury ASA grade was 2 (Table 1). She could walk with a stick before the injury. Plate fixation and cerclage wiring were performed on day 4 after injury (Figure 6). The operative time and blood loss were 107 min and 733 ml, respectively (Table 2). Weight-bearing was limited for six weeks postoperatively. No perioperative complications occurred; however, she could not walk (Table 3) and died approximately one year postoperatively.

**Figure 7:**
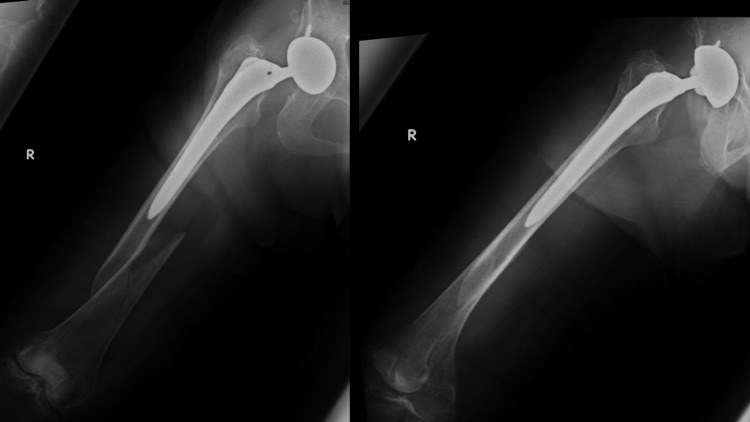
Preoperative radiographs of case 4 Preoperative radiographs of case 4 showed PFF of Vancouver classification Type C1. PFF: periprosthetic femoral fracture.

**Figure 8:**
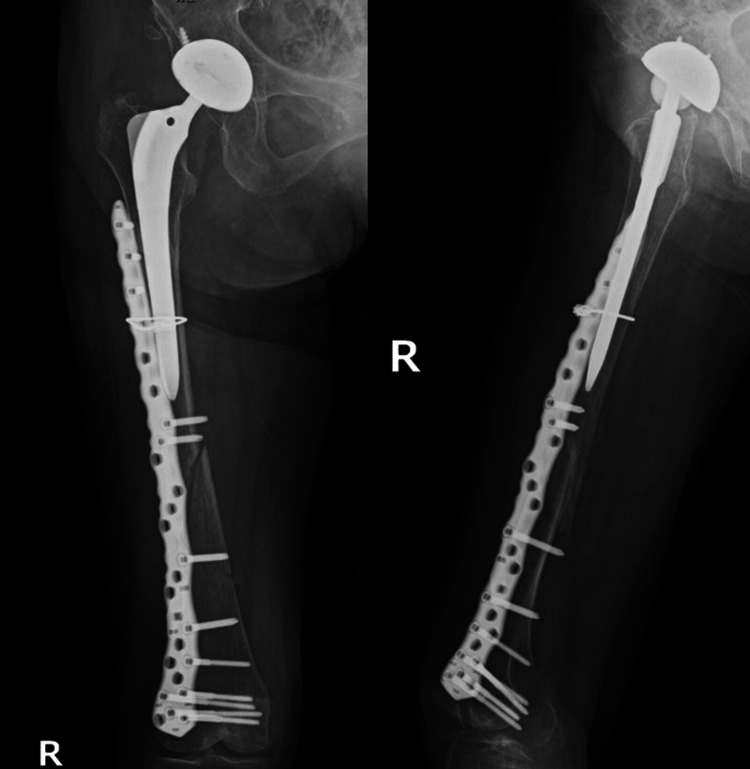
Postoperative radiographs of case 4 ORIF with plate fixation and cerclage wiring was performed. ORIF: open reduction and internal fixation.

Case 4 is a 91-year-old woman who received treatment for hypertension. She had previously undergone THA for osteoarthritis of the hip. Approximately 16 years after the primary surgery, the patient fell and was diagnosed with a Vancouver Type C PFF (Figure 7). The pre-injury ASA grade was 2 (Table 1). She could walk with a stick before the injury. The patient tested positive for coronavirus disease 2019 on admission and underwent surgery more than 10 days after the injury (Figure 8). The operative time and blood loss were 130 min and 697 ml, respectively (Table 2). After four weeks of weight-bearing restriction postoperatively, weight-bearing gait training was initiated. Although she could not regain her walking ability (Table 3), she was still alive at one year postoperatively.

## Discussion

In this study, we investigated the short-term clinical outcomes of ORIF for PPF in very elderly patients aged >90 years. No fatal perioperative complications occurred, and all patients underwent rehabilitation, including gait training, during their stay at our hospital. No patient regained walking ability. No perioperative deaths occurred; however, two deaths occurred within the first postoperative year.

Previous reports showed that the postoperative mortality rates for PFF within one month and one year were high at 9.2% and 22.3%, respectively [12]. Additionally, high ASA grade and age >80 years were reported to be predictors of poor functional outcomes and mortality [13]. The mortality rate within one month was lower in this study than in previous reports; however, the mortality rate within one year was higher. These patients were older than those in previous reports; however, no cases of ASA grade 3 or higher were found. Considering that the current average life expectancy for women in Japan is 87.1 years, the mortality rate within one year postoperatively in our study may not be necessarily high. Additionally, regarding the timing of surgery, a previous study reported that surgery within two days reduced mortality, whereas others reported no effect [10,12]. None of the patients in this study underwent surgery within two days of injury; however, this did not increase perioperative mortality. Although early surgery is ideal, in some cases of ORIF for PFF in very elderly patients, a longer waiting period is unavoidable given the invasiveness and complexity of the procedure. Fortunately in this study, there were no major complications with the perioperative course because the patient's general condition improved during the waiting period.

Previous studies have reported that early weight-bearing decreases the mortality rate [14]. ORIF for PFF requires postoperative weight-bearing restrictions in almost all cases, unlike ORIF for femoral transverse fractures, even if surgery is performed early. These are the limitations of ORIF for PFF. To improve the functional outcomes of ORIF for PFF in very elderly patients, early range-of-motion training of the operated hip, even during the non-weight-bearing period, should be encouraged. In this study, all patients underwent gait training with a walker within the 30-day postoperative period. Some patients were discharged to nursing homes and could not receive continuous gait training with a physical therapist, which may have contributed to the poor clinical outcomes at three months and one year postoperatively.

This study had some limitations. First, it included a small number of patients. Second, some variations were found in the details of the ORIF methods and post-treatments. Third, we did not score hip function or activities of daily living and only assessed gait function and mortality as postoperative clinical outcomes. Finally, because we had no comparison group, we could not show that ORIF improved the prognosis compared to conservative treatment or revision surgery.

## Conclusions

The postoperative ORIF results for PFF in patients aged >90 years showed no fatal perioperative complications and a low mortality rate within 30 days postoperatively. These results suggest that ORIF for PFF can be considered for elderly patients if the preoperative ASA grade is relatively low. Although early surgery could reduce mortality, we believe that adequate preoperative evaluation should be prioritized, particularly for elderly patients.
